# Clinical characteristics of synchronous colorectal cancers in Japan

**DOI:** 10.1186/s12957-016-1027-x

**Published:** 2016-10-24

**Authors:** Takaharu Kato, Sergio Alonso, Yuta Muto, Hiroshi Noda, Yasuyuki Miyakura, Koichi Suzuki, Shingo Tsujinaka, Masaaki Saito, Manuel Perucho, Toshiki Rikiyama

**Affiliations:** 1 Department of Surgery, Saitama Medical Center, Jichi Medical University, 1-847 Amanuma-cho, Omiya-ku, Saitama, 330-8503 Japan; 2 Institute of Predictive and Personalized Medicine of Cancer (IMPPC), Institut d’investigació en ciéncies de la salut Germans Trias I Pujol (IGTP), Campus Can Ruti, 08916 Barcelona, Spain; 3 Sanford Burnham Prebys Medical Discovery Institute, 10901 N. Torrey Pines Rd., La Jolla, CA 92037 USA; 4 Institució Catalana de Recerca i Estudis Avançats (ICREA), Catalan Institution for Research and Advanced Studies, Pg. Lluís Companys 23, 08010 Barcelona, Spain

**Keywords:** Colorectal cancer, Synchronous colorectal cancer, Multiple colorectal cancers, Surgical resection, Colon stenosis

## Abstract

**Background:**

Incidence and clinical characteristics of synchronous colorectal cancer (sCRC) patients significantly vary among studies, likely due to differences in surveillance methodology. If remain undetected, sCRC can progress to more advanced stages seriously aggravating patient prognosis. We studied the incidence and clinicopathological characteristics of Japanese patients with sCRCs who underwent surgery for primary CRC and received exhaustive perioperative surveillance.

**Methods:**

We recruited 1005 patients with surgically resected CRCs between January 2007 and December 2011. The associations of clinical and pathological factors with sCRC development were assessed by univariate and multivariate logistic regression.

**Results:**

Eighty-four patients (8.4 %) developed sCRCs, 16 of them (19.0 %) harboring three or more cancers. Companion sCRCs were smaller and earlier stage than the index lesion (*P* < 0.0001). In multivariate analysis, advanced age (odds ratio (OR) 1.03 per year; *P* = 0.009) and left colon tumor location (OR 1.78; *P* = 0.013) are associated with higher risk of sCRCs, particularly in females. Overall survival did not differ between solitary CRC and sCRC (*P* = 0.62).

**Conclusions:**

Our results highlight the importance of perioperative colonoscopy examination to ensure the absence of sCRCs that, being small and early staged, are more difficult to detect. The incidence of sCRC, and notably of triple or more sCRCs, was higher than previously recognized. Because they are also significantly higher than expected by merely stochastic accumulation of individual cancerous lesions, we suggest that the occurrence of many sCRC reflects a hitherto uncharacterized predisposition condition.

**Electronic supplementary material:**

The online version of this article (doi:10.1186/s12957-016-1027-x) contains supplementary material, which is available to authorized users.

## Background

Colorectal cancer (CRC) is the second and third cause of cancer death in male and female, respectively, in developed countries [1]. Although CRC incidence in Japan and other Asian countries is lower than in western countries, during the last few decades, it has been rapidly increasing, according to recent reports from the World Health Organization [2, 3]. The incidence of synchronous CRC (sCRC) has been estimated to range between 1.1 and 8.1 %, with only small size studies reporting an incidence higher than 4 % [4–20].

Well-established risk factors for sCRC development are familial CRC syndromes, ulcerative colitis, and microsatellite instability (MSI) [13, 21]. A preoperative precise diagnosis of sCRC is essential, because it may influence clinical decision-making regarding the type and extension of the surgical procedure as well as the use of additional treatments. Furthermore, if overlooked, synchronous tumors may require additional surgery and might grow into more advanced stages ultimately leading to the development of distant metastases.

The aim of this study was to investigate the incidence rate of sCRCs in a consecutive series of non-familial Japanese CRC patients and determine the clinical and pathological features of patients developing sCRCs, to estimate the relative contribution of the different risk factors.

## Methods

### Patients

We reviewed 1022 consecutive patients that underwent surgery for primary CRC in Saitama Medical Center, Jichi Medical University, between January 2007 and December 2011. Seventeen cases were excluded from the analysis, including suspected familial adenomatous polyposis syndrome (FAP, *n* = 4), hereditary nonpolyposis colorectal cancer (HNPCC, *n* = 1), patients with history of previous CRC (*n* = 9), and patients with ulcerative colitis (*n* = 3). The remaining 1005 patients were included in this study. Mean patient age was 67.4 ± 11.2 years and mean follow-up interval was 44.3 ± 19.5 months.

In this study, only cancerous lesions histologically proven were considered. Multiple CRC was defined according to the criterion of Moertel et al. [22]: a pathologically proven adenocarcinoma and distinctly separated from the previous line of anastomosis. Cancers diagnosed within 6 months before or after the initial diagnosis were considered as sCRC. In sCRCs, the most pathologically advanced cancer was designated as the index tumor. When more than one tumor were diagnosed with identical pathological stage, the largest one was considered the index tumor and the other lesions were considered the companion tumors.

Colonoscopy was used as standard a preoperative surveillance of the whole colon [23]. Some patients harbored advanced tumors which prevented the advance of the colonoscope to more proximal colon, i.e., impassable stenosis. These patients received an alternative surveillance modality, e.g., 3DCT, barium enema study, colonoscopy after self-expanding metallic stent (SEMS) placement across the obstructing lesion, or intraoperative colonoscopy.

Tumor stage was defined according to the tumor, lymph nodes, and metastasis (TNM) classification of the American Joint Committee on Cancer (6th edition) [24]. Tumor location was classified into three groups as follows: right colon (appendix, cecum, ascending, hepatic flexure, and transverse colon), left colon (splenic flexure, descending, sigmoid, and rectosigmoid junction), and rectum.

### Ethics approval and consent to participate

In this retrospective study, anonymized clinical information from patients from the Saitama Medical Center, Jichi Medical University, was employed. The study was approved by the Research Ethics Committee at Saitama Medical Center, Jichi Medical University, complying with the ethical guidelines of the Declaration of Helsinki [25].

### Statistical analysis

Categorical variables were compared using Fisher’s exact test. Continuous variables were compared using *t* test for those exhibiting a normal distribution, or Wilcoxon-Mann–Whitney test for those that deviated from a normal distribution. Deviation from normality was determined by Shapiro’s test. Differences in survival were studied using Cox’s proportional hazards regression model. Differences were considered statistically significant at *P* < 0.05. The concordance between locations of sCRCs for the same individual was analyzed using Kappa (*K*) statistic. Accuracy was considered poor when *K* was less than 0.20, fair to good when *K* was between 0.20 and 0.40, and good when *K* was larger than 0.40 [15]. All statistical analyses were performed using R (ver.3.1.2.) [26] and OpenEpi web server [27].

## Results

### Clinical and pathological features of sCRCs

**Fig. 1:**
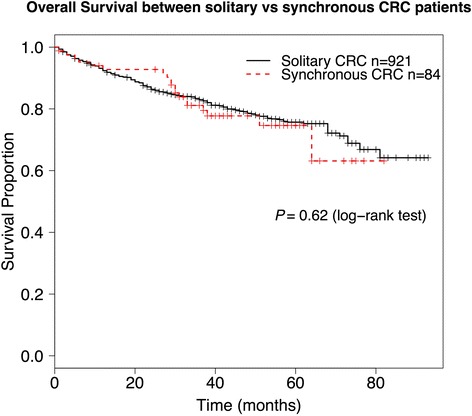
Overall survival of CRC patients with solitary (in *black*) or synchronous CRCs (in *red*) (*P* = 0.62, log-rank test)

Among 1005 patients who underwent surgery for CRC, 84 patients (8.4 %) developed sCRCs (Table 1). Of these, 16 patients (19.0 %) harbored triple or more sCRCs as follows: triple sCRCs occurred in eight patients, quadruple in seven, and quintuple in one. In total, 193 sCRCs were detected in these 84 patients. Table 1 summarizes the differences between patients with solitary CRC and patients with sCRC. Three factors were statistically significant in these univariate analyses: gender (*P* = 0.044), age (*P* = 0.028), and tumor location (*P* = 0.031). Other clinicopathogical features such as tumor size, T stage, tumor differentiation, status of lymph node metastasis status, and frequency of extracolonic malignancies were not different between sCRC and solitary CRC. Overall survival of sCRC patients was also similar to that of solitary CRC patients (*P* = 0.62) (Fig. 1). Multivariate logistic regression analysis using gender, age, and tumor location as explanatory variables confirmed that these three factors independently associated with sCRCs risk (Table 2). When patients were stratified according to gender, both age and tumor location retained statistical significance in women but not in men (Table 2).

**Table 1:** Clinical and pathological characteristics of patients with solitary vs. synchronous CRC

	Solitary CRC	Synchronous CRC	*P* value*
	(921 cases; 93.6 %)	(84 cases; 8.4 %)	
Gender (male/female), no.	575/346	62/22	*0.044*
Mean age, years ± SD	67.1 ± 11.3	70.3 ± 9.5	*0.028*†
Follow-up months ± SD	44.6 ± 19.5	41.6 ± 18.7	0.097†
Location of solitary			*0.031*‡
Right side	308 (33.4 %)	26 (31.0 %)	
Left side	282 (30.6 %)	37 (44.0 %)	
Rectum	331 (35.9 %)	21 (25.0 %)	
Average size; mm ± SD	44.4 ± 25.6	43.8 ± 19.0	0.55†
T factor			0.26†
Tis	32 (3.5 %)	0	
T1	94 (10.2 %)	7 (8.3 %)	
T2	136 (14.8 %)	12 (14.3 %)	
T3	443 (48.1 %)	49 (58.3 %)	
T4	213 (23.1 %)	16 (19 %)	
No residual/uncertain§	3 (0.3 %)	0	
Differentiation			0.25
pap + wel + mod	883 (95.9 %)	83 (98.8 %)	
poor + muc + sig	38 (4.1 %)	1 (1.2 %)	
Lymph node metastasis			0.81
N0	573 (62.2 %)	51 (60.7 %)	
N1/2/3/4	348 (37.8 %)	33 (39.3 %)	
Stage, no.			0.45
0	32 (3.2 %)	0	
I	185 (18.4)	15 (17.9 %)	
II	322 (35.0 %)	31 (36.9 %)	
III	270 (29.3 %)	26 (31.0 %)	
IV	112 (12.2 %)	12 (14.3 %)	
Survival ratio			0.62||
3 years	83.1 %	81.0 %	
5 years	75.7 %	74.5 %	
Extracolonic malignancies			0.85
No	828 (89.9 %)	75 (89.3 %)	
Yes	93 (10.1 %)	9 (10.7 %)	

Values in parentheses are percentages unless indicated otherwise

Italicized data, *P* values <0.05

*Fisher’s exact test

^†^Mann–Whitney test

^||^Log-rank test

^‡^Significance between the rectum and left side

^§^Numbers of no residual/uncertain were not included in the calculation

**Table 2:** Multivariate logistic regression analysis of risk factors for the development of synchronous CRC

Group	Factor	Odds ratio (95 % CI)	*P* value
All patients (*n* = 1005)	Gender (male)	1.67 (1.02–2.84)	*0.047*
	Age	1.03 (1.01–1.05)	*0.009*
	Location (left)	1.78 (1.12–2.81)	*0.013*
Men (*n* = 637)	Age	1.02 (0.99–1.05)	0.16
	Location (left)	1.60 (0.93–2.72)	0.08
Women (*n* = 368)	Age	1.06 (1.02–1.11)	*0.010*
	Location (left)	2.62 (1.05–6.41)	*0.035*

In italics, *P* values <0.05

### Incidence of sCRCs is not explained by stochastic accumulation of lesions

The incidence of sCRC among patients (84 in 1005) was more than twofold higher than predicted by purely stochastic accumulation of cancerous lesions considering the reported CRC incidence in the Japanese population [28], after adjusting by age and gender (standardized incidence ratio (SIR) = 2.2; confidence interval (CI) = 1.75–2.69; *P* = 2.1 × 10^−10^). Moreover, among the 84 sCRC patients, 16 had 3 or more sCRC, an incidence 2.3-fold higher than expected under the assumption that sCRC lesions would occur independently with the probability of 0.084 per cancer (expected = 7; CI = 2–13; binomial distribution; *P* = 9.4 × 10^−4^). A more refined analysis adjusting by age (in 10-year bins) and gender confirmed a 2.2-fold higher risk of additional companion carcinomas in patients that already harbored one index sCRC (SIR = 2.16; CI = 1.43–3.14; *P* = 6 × 10^−4^).

### Incidence of sCRC in older vs. younger patients

Familial CRC has been associated with a higher incidence of sCRC [13]. Self-reported familial cases were excluded from our analyses (see the “Methods” section). However, the proportion of HNPCC cases among the initially recruited 1022 patients was very low (*n* = 1, 0.1 %), even for the Japanese population [8]. In the self-reported 1005 non-familial cases included in the analysis, there were 73 patients (7.3 %) younger than 50 years (Additional file 1: Figure S1), a few of which could correspond to unreported cases with family history of cancer. To assess whether the inclusion of these patients might be associated with the high incidence of sCRC found in our study, we compared the sCRC incidence in patients older vs. younger than 50 years. Notably, sCRC incidence was higher in older (83/932, 8.9 %) than in younger (1/73, 1.4 %) patients (odds ratio (OR) = 7.03; CI = 1.2–284.9, *P* = 0.02). Similar results were obtained when cutting at a higher age threshold of 60 years: 75/786 (9.5 %) in older vs. 9/219 (4.1 %) in younger patients (OR = 2.46; CI = 1.2–5.7; *P* = 0.008).

### Diagnosis and treatments of patients with sCRCs

The vast majority of the sCRCs were diagnosed preoperatively (*n* = 185, 95.9 %), either by standard preoperative colonoscopy (*n* = 179, including the 84 index lesions and 94 synchronous lesions), by barium enema (three tumors in two patients), 3DCT (two tumors in two patients), or by intraoperative colonoscopy (one tumor in one patient). SEMS were placed in six stenotic patients, but none of them were found to harbor sCRCs. Only eight sCRCs (4.1 %) were incidentally identified by postoperative pathological analysis in four patients (Table 3).

**Table 3:** Pathological findings, surveillance, and treatment methods of synchronous CRC patients

	Index	S2	S3	S4	S5
Number of patients	84	84	16	8	1
Location of tumor					
Right side	26 31.0 %)	31 (36.9 %)	3 (18.8 %)	1 (12.5 %)	1
Left-side	37 (44.0 %)	37 (44.0 %)	10 (62.5 %)	7 (87.5 %)	0
Rectum	21 (25.0 %)	16 (19.0 %)	3 (18.8 %)	0	0
*P* value		0.58	0.47	0.081	
Average size, mm ± SD	43.8 ± 19.0	23.9 ± 15.1	18.0 ± 8.3	14.9 ± 1.5	15
*P* value*		*<0.001*	*<0.001*	*<0.001*	N.A.
T stage					
Tis	0 (0 %)	39 (46.4 %)	11 (68.8 %)	6 (75 %)	1
T1	7 (8.3 %)	23 (27.4 %)	4 (25 %)	1 (12.5 %)	0
T2	12 (14.3 %)	8 (9.5 %)	1 (6.25 %)	0	0
T3	49 (58.3 %)	10 (11.9 %)	0	0	0
T4	16 (19 %)	1 (1.2 %)	0	0	0
No residual/uncertain^a^	0	3 (3.6 %)	0	1 (12.5 %)	0
*P* value		*<0.001*	*<0.001*	*<0.001*	N.A.
Histological type					
pap + well + mod	83 (98.8 %)	81 (96.4 %)	14 (87.5 %)	6 (75 %)	0
poor + muc + sig	1 (1.2 %)	0	0	0	0
Others/no residual/uncertain†	0	3 (3.6 %)	2 (12.5 %)	2 (25 %)	1
*P* value		1	1	1	N.A.
Diagnosis methods					
Endoscopy (pre-op)	84 (100 %)	75 (89.3 %)	12 (75 %)	7 (87.5 %)	1
3DCT	0	2 (2.4 %)	0	0	0
Barium enema study	0	2 (2.4 %)	1 (6.25 %)	0	0
Endoscopy (intra-op)	0	1 (1.2 %)	0	0	0
Post-op pathologically	0	4 (4.8 %)	3 (18.8 %)	1 (12.5 %)	0
Treatment methods					
Endoscopic resection	0	24 (28.6 %)	6 (37.5 %)	5 (62.5 %)	1
Normal surgery	69 (82.1 %)	45 (53.6 %)	9 (56.3 %)	2 (25 %)	0
Extended surgery	15 (17.9 %)	15 (17.9 %)	1 (6.25 %)	1 (12.5 %)	0

Values in parentheses are percentages unless otherwise indicated

*P* values were calculated comparing to index tumor using Fisher’s exact test except for *Mann–Whitney test

In italics, *P* values <0.05

^a^Numbers of others/no residual/uncertain were not included in the calculation

Secondary, tertiary, or quaternary sCRCs were significantly smaller and less advanced than the index tumor (Table 3). sCRCs tended to occur in the same surgical segment, with a fair to good association (*K* = 0.30, *P* = 0.0001) (Table 4). However, 43 patients (51.2 %) developed sCRCs in different surgical segments.

**Table 4:** Location of index and companion synchronous CRCs

	Companion synchronous tumor location			
Index tumor location	Right colon	Left colon	Rectum	*K* ^a^
Right colon (*n* = 26)	17	11	2	
Left colon (*n* = 37)	10	35	8	
Rectum (*n* = 21)	8	9	9	0.30

^a^Unweighted Cohen’s kappa: *P* = 0.0001

sCRCs were treated by endoscopy (36 tumors in 24 patients), by standard surgery (125 in 69 patients) or by extended surgery (32 tumors in 15 patients). All patients in which endoscopical resection of the companion sCRCs was performed subsequently underwent standard surgery for the resection of the index lesion (Table 3). We found no difference in survival between patients who underwent extended surgery compared to patients with standard surgery (*P* = 0.91).

### Examination and treatment of patients with impassable stenosis

In this series, 139 patients harbored locally advanced tumors in the left colon or rectum narrowing the lumen and preventing the passage of the colonoscope (impassable stenosis), thus hampering the detection of possible sCRCs developing at more proximal locations. Of these, 54 patients received 3DCT analysis and one synchronous tumor was detected, 53 patients underwent barium enema study and two synchronous tumors were found, four underwent colonoscopy after SEMS placement across the obstructing lesion and no synchronous tumors were found, and five underwent intraoperative colonoscopy and one synchronous tumor was found. The other 23 patients could not receive any additional analysis of the proximal colon. In these patients, three synchronous tumors were incidentally found by pathological reports after surgery. In total, only four of the 139 (2.9 %) distal-stenotic CRC patients developed sCRCs in the proximal colon.

## Discussion

The incidence of sCRC (8.4 %) in our group of 1005 CRC patients is higher than that found in most of previous series [4–20] and significantly higher than expected by stochastic accumulation of cancerous lesions considering the incidence of CRC in the general population of Japan (SIR = 2.2; CI = 1.75–2.69; *P* = 2.1 × 10^−10^). In this regard, our data is consistent with the long-standing but still unresolved observation that cancer patients are at higher risk of developing second independent malignancies that cancer-naïve individuals [29]. Moreover, the incidence of triple or more sCRC patients in our study (16 in 1005, 1.6 %) was significantly higher than previously reported (0.1–0.7 %, *P* = 9.6 × 10^−4^) [9, 10, 13–15, 18] and 2.2-fold higher than expected by stochastic accumulation of independent cancers. Thus, patients who developed one sCRC were at increased risk of developing additional synchronous malignancies. The higher propensity to develop independent cancers in cancer patients compared with the general population, and within them the existence of patients with even higher propensity to multiple CRCs, further supports that genetic and environmental risk factors, and not only stochastic molecular mechanisms, underlie cancer susceptibility [30].

Age at diagnosis was an independent risk factor for the occurrence of sCRC when considering men and women together (Table 2). This observation is also in agreement with most previous reports [15, 17, 19, 20]; however, some reports did not find an association, or even found a reverse association, between age and synchronous CRC development [9, 11]. Notably, when stratifying the patients according to gender, we found that age was a stronger risk factor in women than in men (Table 2). To the best of our knowledge, no previous report mentioned this gender disparity in the association between age and sCRC risk.

CRC patients with family history of cancer have a higher predisposition to develop sCRC [13]. In our study, all patients with self-reported or diagnosed familial syndromes were excluded (FAP or HNPCC, see the “Methods” section). However, the information regarding family history was essentially based on self-reported, possibly inaccurate, testimonials from the patients. Due to the retrospective design, we could not obtain more detailed and accurate information. The incidence of confirmed HNPCC in our series was 0.1 %, which is low even when considering that the incidence of HNPCC has been reported to be as low as 0.4 % in Japan [8], certainly much lower than in North American or European populations (1–3 %) [8, 31, 32]. It is therefore possible that a few individuals classified as non-familial cases were actually undiagnosed HNPCC patients.

We then analyzed the incidence of sCRC in patients older vs. younger than 50 years, taking into account that most HNPCC patients develop CRC before that age [31, 33]. Notably, the incidence of sCRC was higher in older patients (OR = 7.03; CI = 1.2–284.9, *P* = 0.02). In addition, HNPCC tumors preferentially develop in the right side colon, but in our study, most of the sCRCs (68.4 %) developed in the left colon or rectum. Taking these observations together, it seems unlikely that the high sCRC incidence found in our series was due to inadvertently inclusion of familial CRC patients in the study.

Previous studies reported that sCRC develops in the right colon more frequently than solitary CRC, although this association has not been confirmed in other studies [7, 12]. In our series, we found no difference in the frequency of solitary vs. synchronous CRCs developing in the right colon. However, we found a higher incidence of sCRCs in the left colon (comprising descending and sigmoid colon) and a lower incidence in the rectum (Table 1). In our series, sCRCs often developed closely to each other, facilitating in some cases clinical and pathological detection (Table 4). However, 43 patients (51 %) harbored sCRCs in separate surgical segments, a proportion that is in line with previous findings (43 to 78 %) [6, 34]. In 28 of these patients, sCRCs could be resected by endoscopy combined with standard surgery. In the remaining 15 patients, however, extended surgery was performed to resect all the lesions.

We found no significant differences in size or stage between the sCRC index lesions (the larger and more advanced among the sCRCs) and solitary cases (Table 1). On the other hand, the companion sCRCs were smaller and less advanced than both the index lesions (Table 3) and the solitary tumors, in agreement with previous reports [5, 9, 16, 34]. Small or early CRCs are more likely to be overlooked in the preoperative surveillance [35, 36]. Some of the tumors found during the postoperative follow-up (metachronous) might be in fact overlooked sCRCs, although it is difficult to distinguish metachronous and sCRC with precision [37].

A common reason why synchronous lesions may be missed is impassable stenosis due to large tumors in the distal side of colon, preventing the lesions in the proximal colon from being examined [7]. If a patient cannot undergo complete examination of the large bowel before the surgery, colonoscopy analysis is required during, or as soon as possible after surgery: otherwise, the overlooked tumors might advance and reach an unresectable status. Among 1005 cases, total colonoscopy could not be performed in 227 patients (22.6 %) due to impassable stenosis. This was not a serious concern when occurring in the right side colon (*n* = 88), because the whole proximal colon would be resected during the standard surgical right hemicolectomy. When the advanced lesion was located in left colon or rectum (*n* = 139), however, the patients underwent other modality of surveillance to decide the most appropriate surgical treatment. Among these patients, sCRCs were detected in the proximal colon of five patients: in two cases by using 3DCT, one by barium study, one by intraoperative colonoscopy, and one was incidentally detected in the post-surgery pathological analysis. Since there are some difference of detection rate among 3DCT, barium enema, and colonoscopy [38], we confess the possibility that some sCRC might have been missed among the patients with impassable stenosis.

The prognosis of patients with sCRC, compared with patients with solitary CRC, is unclear. It has been documented to be better, the same, or worse, depending on the study [15, 39]. This variation is likely caused by differences in sample size, length of follow-up, and other factors such as different proportion of advanced vs. early tumors and, therefore, needs to be interpreted with caution. In our study, we found no difference in tumor size, stage, differentiation, or in survival rates between solitary and sCRC patients (Table 1). The most important complications associated with sCRC in comparison with solitary CRC derive mainly from the higher propensity of these patients to develop metachronous tumors [40] as well as the possibility of overlooking small or difficult to access synchronous lesions that might later develop into more advanced cancers.

## Conclusions

We show that the sCRC incidence is higher than that of most previous reports. Triple or more sCRCs were also detected more frequently than in previous studies. We studied a relatively large consecutive series of CRC patients that, in contrast with other reports, underwent throughout perioperative examination by several complementary methodologies to minimize the accidental overlooking of sCRC lesions. Since false positives are absent in our study, as all identified lesions were histologically proven, it seems more likely that the lower frequency reported is due to the presence of false negatives in some previous studies. Ethnic factors may also influence the actual incidence differences, and a further examination of this issue is warranted.

Our results strengthen the importance of a thorough, extensive examination to avoid overlooking small or early-staged synchronous lesions. When the locally advanced tumor narrows the lumen and prevents the passage of the colonoscope, it is recommended to analyze the proximal colon by other methodologies in order to decide the most appropriate surgical procedure. Patients that cannot undergo complete colon surveillance need to be studied as soon as possible after surgery to rule out the existence of sCRCs.

Our results are also valuable at a more fundamental level, showing that the sCRC incidence cannot be explained just by the stochastic accumulation of individual cancerous lesions and consequently of the underlying somatic cancer-driving genomic alterations. We conclude that this can be explained by the existence of uncharacterized underlying genetic, environmental, or both, susceptibility factors for multiple CRC.

## Additional file

Additional file 1: Figure S1. Age distribution of the CRC patients in the 1005 cases consecutive series recruited for this work. Blue line: men. Red line: women. Seventy-three patients were younger than 50 years (left of the dashed line), and 932 patients were older than 50 years. (PDF 91 kb)

## Abbreviations

CI: Confidence interval
CRC: Colorectal cancer
FAP: Familiar adenomatous polyposis
HNPCC: Hereditary nonpolyposis colorectal cancer
MSI: Microsatellite instability
OR: Odds ratio
sCRC: Synchronous colorectal cancer
SEMS: Self-expanding metallic stent
SIR: Standardized incidence ratio
