# Supplementary material for: Clinical characteristics of synchronous colorectal cancers in Japan
Source: World J Surg Oncol. 2016 Oct 24;14:272. doi: 10.1186/s12957-016-1027-x (PMC5078884; doi:10.1186/s12957-016-1027-x)
Supplement: Additional file 1: Figure S1. — Age distribution of the CRC patients in the 1005 cases consecutive series recruited for this work. Blue line: men. Red line: women. Seventy-three patients were younger than 50 years (left of the dashed line), and 932 patients were older than 50 years. (PDF 91 kb) [file 12957_2016_1027_MOESM1_ESM.pdf]

Clinical characteristics of synchronous colorectal cancers in Japan

Takaharu Kato, M.D., Ph.D., Sergio Alonso, Ph.D., Yuta Muto, M.D., Ph.D., Hiroshi Noda, M.D., Ph.D., Yasuyuki Miyakura, M.D., Ph.D., Koichi Suzuki, M.D., Ph.D. , Shingo Tsujinaka, M.D., Ph.D. , Masaaki Saito, M.D., Ph.D., Manuel Perucho, Ph.D., and Toshiki Rikiyama, M.D., Ph.D.

Supplementary information

Figure S1

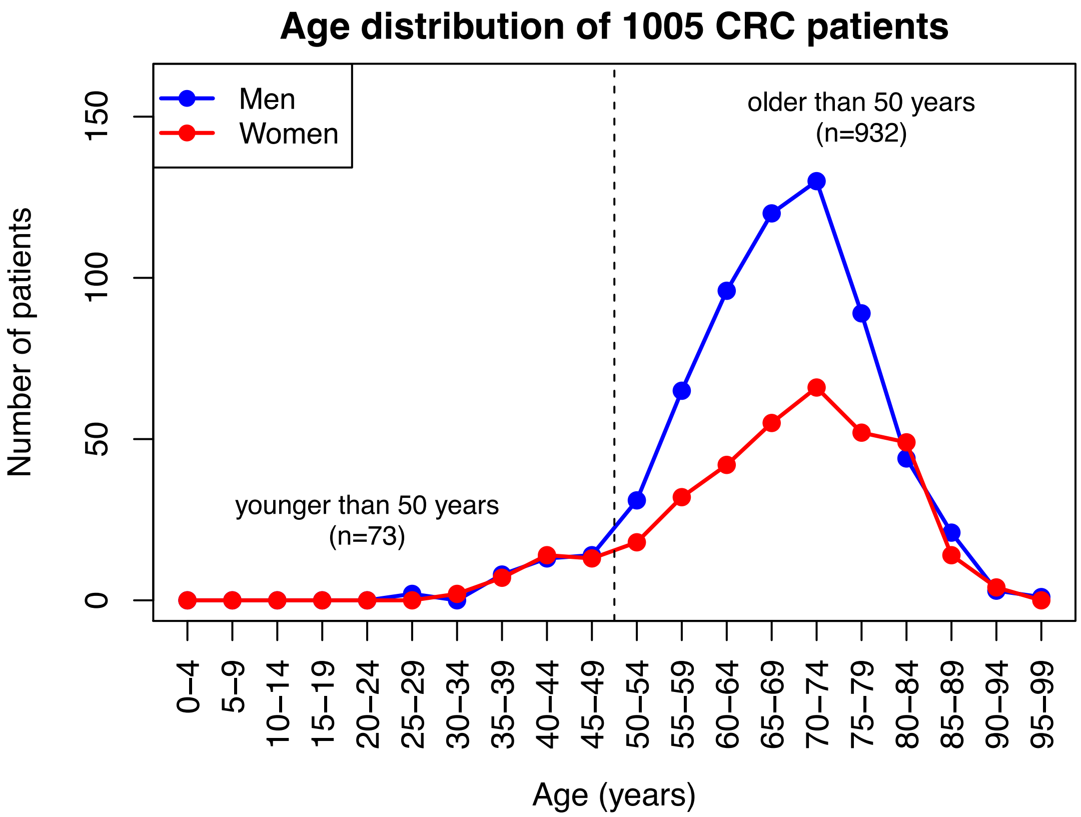

Figure S1. Age distribution of the CRC patients in the 1,005 cases consecutive series recruited for this work. Blue line: men. Red line: women. Seventy three patients were younger than 50 years (left of the dashed line), and 932 patients were older than 50 years.
